# Discourses Delivered by Appointment before the Cincinnati Medical Library Association, January 9th and 10th, 1852

**Published:** 1852-03

**Authors:** 


### REVIEWS.

---

Art. IV.—*Discourses delivered by appointment before the Cincinnati Medical Library Association*, January 9th and 10th, 1852. By Daniel Drake, M. D. Cincinnati: Moore & Anderson. 24 mo. pp. 93.

The Physicians of Cincinnati recently formed an Association for the purpose of collecting a medical library, and invited Dr. Drake to deliver an address before them on the occasion of opening their reading rooms. What was intended, at first, to be "nothing more than a brief inquiry into the origin, progress, and influence of the periodical literature of our profession," has grown into the Discourses before us, the mind of the eloquent author naturally warming with themes so fnll of pleasing reminiscences. As he compared the present expansion and power of American medical literature with what it was half a century ago, he says, "the aspects of the little village and its infant profession rose so vividly before him, that he could neither banish or keep from looking at them." The result was, that he resolved to give, as a preliminary discourse, a sketch of the early physicians, and of the scenery and society of Cincinnati.

We are glad that this occasion offered, and esteem it a fortunate circumstance that its commemoration was committed to one so familiar as the author of these discourses with our early professional history. The scenes which he describes are such as never can be witnessed again on our continent. The settlement of the Western States took place under circumstances the like of which can never recur even in our most distant territories. Steam has had the effect to lengthen time and shorten space in such a way, that the emigrants to California and Oregon are not so far removed from us as were the first settlers of Kentucky and Ohio from the cities of our Atlantic seaboard. Not only can they perform the immense journey in less time, but they are able to carry with them to their distant homes all the comforts and luxuries that commerce affords. They form new colonies, but are surrounded at at once with all the appliances and arts of the highest civilization. All that the skill of Paris or Birmingham can produce—the products of the Indies and the ice of Boston—mechanics of every trade, lawyers, preachers, printers, physicians, are transported to them with a celerity of which the navigators of former times had no conception. Whatever is discovered in science or the arts, whatever is done in war, politics or fashion, in any quarter of the world, is borne to them, not indeed "upon the wings of the wind," but by steamers that in fleetness surpass the wind. Wherever they fix their abodes they find comparative peace and security, for the terror of our national name now affords protection from all the savage tribes.

How different was the case with our forefathers! Isolation, almost complete, from the old settlements; a handful of men, with their women and children, shut up in forts, and in continual danger of the Indian's tomahawk and scalping knife; with none of the luxuries and few of the comforts of life: such was the condition of those who first came to the West.

And Medicine, what was it at that early day? There was then but one medical school of note in the country, and but a single medical journal. A student who would attend lectures at that time must be possessed of ample means, and the result was that among the practitioners of that period there were but few graduates in medicine. Medical books were published at long intervals in this country, and were consequently scarce and expensive; and the few that were accessible to the practitioner in the backwoods were but poor allies, we should say, in his contests with disease. His supply of medicines was almost as scanty and quite as precarious as the equipment of his library; and nurses, and the comforts of the sick-chamber which so much aid the operation of our remedies, it may be said there were none.

There is a peculiar fitness in Dr. Drake's writing the medical history of the chief city of the West. A citizen of the West for more than half a century, he has watched the progress of his profession in that city for a longer period than falls to the lot of most practitioners to be engaged in its active duties. He is the only remaining one of the early physicians of Cincinnati, and stands as the connecting link between two generations of medical men. In preparing these discourses, the author had a far more serious object in view than to subserve the interests of the association for which they were prepared, or illustrate the early history of the city to which he is so warmly attached—he aimed at conferring a lasting benefit upon the profession. He would render our profession *national.* He would save it from the calamity of becoming narrow, sectional, bigoted, to which the very structure of our admirable government gives it an unhappy tendency. He would have every American physician to feel that he is one of a great, catholic brotherhood, as wide as our vast territory, and bound together by the interests of a common science. With this feeling his discourses were composed, and in this spirit he dedicates them to Prof. Dickson, of South Carolina, remarking:

I might find, either in my personal obligations to you, or in your labors as a teacher and writer, a very sufficient reason for some public expression of respect; but by inscribing to you this little work on the medical and social history of a new city so remote from the old and classical metropolis of the South, I wish to manifest what I feel and think, that neither difference of age, nor distance of place, should be permitted to break up the unity, integrity and kind feeling of our beloved, national profession.

In most other countries, the tendency of the medical profession, as of all things else, is to centralization. France and England have physicians everywhere, but they have no medical profession out of Paris and London. The metropolis swallows up everything. Dublin is to Ireland, and Edinburgh is to Scotland, what London and Paris are to England and France—the fountain-heads of medical science. They give character and unity to the profession, which there, as in all the States of Europe, is eminently national.

We do not insist that this is the most desirable condition of things; we do not deny that there are disadvantages connected with it; but, whatever its merits or demerits, it can never obtain in our country. We are divided into many States, and each commonwealth has its medical emporium. The States are again subdivided into counties, and many times it must happen that more than one town in a State will claim to be the metropolis. We are, moreover, divided by nature into various great regions, and these geographical lines are opposed to consolidation. All the tendencies with us, in a word, are in the opposite direction; nor are there wanting those who foster and encourage a sectional spirit. But to the credit of the periodical literature of our profession, be it said, but little countenance has been given to this spirit by our journals. With most commendable unanimity they have discouraged and rebuked those mean local jealousies, which would break up our national profession into fragments, and array the physicians of different sections against each other in all the bitterness of partisan warfare. A few have shown favor to sectional medicine. One, here and there, has insisted upon medicine's being studied where it is to be practiced. Now and then, the sentiment has been advanced, that medical science is bounded by zones, and that the teachings of the North are of no avail in the South—that there are peculiar institutions, and peculiarities of climate, soil, and water, in every section, which render it imperative that its practitioners receive their medical education at home. But, for the most part, this ultraism has been scouted by the medical press. It had its origin, we believe, somewhere in the valley of the Mississippi; it has not traveled beyond it, and for the credit of the profession, let us hope that it is coming to an end, and will soon be heard of no more.

But we must pass from these general topics suggested by Dr. Drake's address, and take up the thread of history furnished by his interesting pages.

It was on the 26th of December, 1788, that *Israel Ludlow* and his associates from New Jersey escaped from the floating ice of the Ohio, and reared their half-faced camps on what is now the site of Cincinnati. In twelve years the population did not amount to one thousand, and was only two thousand three hundred and twenty, in the year 1809, twenty-one years after its settlement. Of the first physicians of the city the biographical memorials to be collected at this late day are brief and scanty. The pioneers of the profession were chiefly the surgeons of the army. They appear to have been benevolent men, and often polished and elegant gentlemen of scholarly attainments, but generally were not graduates in medicine. It was their custom not merely to give gratuitous attendance on the people of the village, but also to furnish medicines from the army hospital chests, through a period when none were imported from the east. We quote Dr. Drake's brief notices of these departed worthies:

The first of these surgeons, as far as I can learn, was Dr. Richard Allison, who remained after the army was disbanded, and, therefore, I shall speak of him hereafter, with those in civil life.

Another was Dr. Adams, of whom I only know, that he belonged to the state of Massachusetts, to which he returned. I cannot learn at what period he was stationed here, but hope this reference to him, may bring us some biographical facts.

Nor have I been able to fix the time or times, when Dr. John Carmichael lent his aid to the infant settlement. From a highly intelligent lady,* I learn that in the year 1802, when a reduction of the army took place, he was among the discharged; and being with Gen. Wilkinson, at Fort Adams, below Natchez, was employed to transport to New Orleans the baggage and munitions of the garrison, which took possession of Louisiana after its purchase from France. With the proceeds of this enterprize, he became a cotton planter in Mississippi territory, acquired wealth, and lived to an advanced age.

Dr. Joseph Phillips was one of the surgeons, who is still remembered for his knd offices. The venerable relict of the late Gen. John S. Gano, (the intrepid surveyor of the route pursued by St. Clair's army) has, within the last few days, informed me, that on the suggestion of Gen. Harrison, Dr. Phillips was brought in from Fort Hamilton, to rescue her husband from the hands of a couple of quacks. She remembered him as a physician of skill and gentleman of much personal presence. From his namesake and distant relative, Mr. H. G. Phillips of Dayton, I learn that he was a native of Lawrenceville, New Jersey; that he came out with Wayne's army; and, after the treaty of peace, returned to his birth-place. Resuming his practice, he lived much respected both as a physician and citizen, till his death; which took place only five or six years since, when he was eighty years old or upward. He probably was the last to die, of all the early members of our profession; and one feels a sort of surprise, at learning that a physician who practiced in Cincinnati when it was

---

*Mrs. Col. Strong.

a mere encampment, should have been alive so near the present time.

Dr. John Sellman, like Dr. Allison, remained after he left the army, and must be spoken of with the physicians in civil life.

Dr. John Elliott came out with St. Clair. He was stationed here at various times. He was disbanded with the regiment to which he belonged, in the year 1802. His two daughters were then in this place, but he did not settle here. The elder married the late Hon. Joseph H. Crane, who removed to the new village of Dayton. In the summer of 1804 I saw the Doctor there, a highly accomplished gentleman, with a purple silk coat, which contrasted strangely with the surrounding thickets of brush and hazle bushes. He resided in Dayton till his death, which took place in 1809. New York was his native State.

The last of the army surgeons of whom I have been able to collect any account, was Dr. Joseph Strong. He came out with General Wayne in the spring of 1793; was with him in the battle of the next year; and also attended the Greenville treaty in 1794. Dr. Strong was a native of Connecticut, and a graduate of Yale College in the arts, but, like all whom I have named, not in Medicine. He returned to the east about 1795, and settled in Philadelphia, where he made the acquaintance of Dr. Rush, and acquired a respectable practice, but died in April, 1812, at the age of 43 years. From his daughter Mrs. Col, Bond of our city, I have obtained two of his manuscripts; which, as the only literary remains of the military branch of our profession, must not be passed over in silence.

The first, in his own hand-writing, is dated at Fort St. Clair, "August 22nd, 1793." The beginning is lost, but it appears to have been a discourse on the professional and moral duties of the physician. It abounds in figures of speech, drawn from the army; and while its style indicates the young man, its judicious advice would do credit to the old."

---

The late President Harrison is to be ranked among the early army surgeons of Cincinnati. He studied medicine, and attended a course of lectures in the University of Pennsylvania; but his military taste prevailed over his love of medicine, and he entered the army, not as a surgeon, but as an officer of the line.

His professional knowledge, however, enabled him frequently to afford relief to those who could not, at the moment, command the services of a physician, and also inspired him with an abiding interest in the progress of the profession. This he successfully displayed more than twenty-five years afterwards, when a member of the Senate of Ohio. The bill for establishing the Commercial Hospital and Lunatic Asylum of Ohio, met with much opposition, against which he exerted himself with his usual characteristic energy; and I shall ever remember seeing him enter the Senate Chamber, with several books of reference under his arm, to make a speech, in which he instructed the Senators concerning hospitals, and their value to Medical Schools, without which the people of the State could not have good physicians. As you are all aware, the hospital was founded. The Legislature had chartered the Medical College of Ohio the year before, and at a subsequent time, he acted as one of its trustees.

This completes the catalogue of the army surgeons, and Dr. Drake next takes up the civil history of the profession, and though the annals are very brief we have found them interesting, and believe they will prove so to our readers. We copy his account of the earliest medical settlers:

Dr. William Burnet, Jun., as far as I can learn, was the first physician, apart from the army surgeon, who emigrated to Cincinnati. He arrived in 1789, but a few months after the first landing, bringing with him both books and medicines. There was but little, then, to do in the profession, and he spent a part of his time at North Bend, with the great proprietary of the Miami country, John Cleves Symmes. In the spring of 1791, as I learn from his brother, the venerable Judge Burnet, he revisited his native state, but with the intention of returning here for permanent residence, and the practice of his profession. Soon after reaching there he obtained from the Grand Masonic Lodge of New Jersey, the warrant under which the Nova Cesarea Harmony Lodge, No. 2, of this city, was constituted; and by the same authority was appointed its first Master; but the death of his father soon afterwards, prevented his coming back. He subsequently lived near Newark in that state, but I can not tell when he died. Dr. Burnet, like Dr. Strong, was a gentleman of classical education, but not a graduate in medicine. He had been a surgeon's mate in the revolutionary army, under his father, who was Surgeon General. In going from here, he left his books behind him, and I hope to be able to place one or more of them on the shelves of our library, where they ought to be preserved as the first ever brought to the city.

Dr. Calvin Morrell, of the same state, was appointed Warden of the projected Lodge, at the time Dr. Burnet was appointed Master. Whether he had come out with Dr. Burnet, or emigrated afterwards, is uncertain. I find, by the records, that he was present at the organization of the Lodge, December 27, 1794, just six years after the settlement of the town. Soon after this, he moved thirty miles back into the country; and ultimately became united with the Shakers of Union village, near Lebanon, where I believe he died. From all I have been able to learn, he did not do much business here, nor make any lasting impression on the little community.

Dr. John Hole arrived here in 1790 or 91. He probably came before Dr. Morrell. I do not know his native place. In the winter of 1792–3, he practiced inoculation here, and also in the village of Columbia, the smallpox having been then introduced for the first time. He was not a man of much education or social rank; and before the end of the Indian wars, 1794, he left the town. I have not learned of what State he was a native, nor when or where he died.

Dr. Drake suppresses the name of one of the pioneer physicians who, on an alarm of Indians spread by some wag, ran away and came over into Kentucky. As a faithful historian, we think he was bound to give the name of the recreant, however discreditable to the profession.

The next immigrant physician was Dr. Robert McClure, whose wife, as I was lately told, by an aged and accurate matron, was a lady of such excellent kindness of heart, that she greatly commended him to the people; a biographical fact which it may be well for the younger members of our Association to treasure up. Dr. McClure came from Brownsville, Pennsylvania about 1792. In 1801 he moved into the country, and subsequently returned to his native place, where he died. His residence was on Sycamore, between Third and Fourth streets. He does not seem to have been a man of great education; but did a respectable business. Our aged people relate that in those days it was customary with the officers of the army, to drink bitters in the morning—those of *Dr. Stoughton* of London being preferred; but as importations were sometimes suspended, Dr. McClure made a tincture, and putting it up in small vials, labelled them—"Best Stoughton's bitters, prepared in Cincinnati by Dr. Robert McClure." The solecism seems to have been quite an occasion of merriment with the officers of the army. We see from this anecdote, that a business which has since been so profitable to certain persons in our city, was begun in the days of its early infancy.

In the next paragraph Dr. Drake, after mentioning the name of his preceptor, goes on to describe Cincinnati as it was fifty years ago. We are compelled to omit his description for the sake of finding room for his biographical sketches. What he has said of the earliest physicians he collected from others; what follows, except the details respecting the army surgeons, Allison and Sellman, is chiefly furnished by his own recollections. After quitting the army these gentlemen settled in Cincinnati, and with Dr. Cranmer and Dr. Goforth constituted the whole Faculty of Cincinnati in the first year of this century. In fact, as Dr. Allison had retired into the country the number of practicing physicians was really but three; and yet "the proportion to the population," adds Dr. Drake, "was even then greater than it is at present."

We extract his account of Dr. Allison who first exercised the healing art in Cincinnati:

Dr. Richard Allison was born near Goshen, State of New York, in 1757. He was not a graduate in medicine, and I can say nothing of his early opportunities. In 1776, he entered the army of the Revolution as a surgeon's mate, and continued in it to the end of the war. Where he then practiced his profession I have not been able to learn. When the general government was about to raise and send an army into the west, he re-entered the service, and acted as Surgeon General in the campaigns of Harmer, St. Clair, and Wayne. I am not in possession of documentary evidence, on the manner in which he discharged the responsible and trying duties of his station in the army. They were highly responsible, because the life of every soldier was precious to the insulated families on both banks of the river; and equally trying, because the hospital stores, medicines and instruments were scant and imperfect. If he had not acquitted himself successfully, it is not probable he would have been kept in his position. He must, of course, have performed many operations, after the several battles, but kept I believe no record of them. In St. Clair's defeat he was greatly exposed; for he was obliged to leave the wounded and mingle in the fight. His horse received a bullet in the head. It remained imbedded in the skull; and when riding him through the village in after times, he would jocosely remark, that his *horse* had more in his head than some *doctors* he had known. Whenever stationed here he gave such assistance to the people of the village, as made him a general favorite; and after his resignation many of them employed him, when his services were no longer gratuitous.

After the campaign of St. Clair in 1791, the greater part of the army, consisting of militia and volunteers returned home, and the few regulars were distributed as they were most needed for the defense of the new and weak settlements on both banks of the Ohio. At that time there was a military station where the lower part of Jeffersonville, Indiana, now stands. It was called Fort Finney. The foundations of it are still to be seen, opposite the city of Louisville. In the summer of 1792, between the campaigns of St. Clair and Wayne, Dr. Allison was stationed at this post, and it appears from a fact I am about to mention, that the people of Louisville and its vicinity, were, at that time, not less dependent on the army surgeons, than those of Cincinnati. In the year 1782, Col. William Pope, of Virginia, emigrated to Louisville, (better known as the Falls, or the mouth of Bear Grass) where he reared a family, several of whom were more or less distinguished in the first quarter of this century. The most conspicuous was his son John, known throughout the Union as the eminent Kentucky statesman with one arm. In 1792, Col. Pope resided in Sulivan's station, six miles out of Louisaille; where one of the settlers had constructed a sort of mill, for crushing Indian corn stalks; from the juice of which, the people made molasses, and also a kind of beer. Young John, at that time 14 years old, was engaged in *feeding* this mill with stalks, when his hand was caught, and his arm crushed nearly to the shoulder. Dr. Allison was sent for to Fort Finney, and amputated the limb. The talents and heroism of his young patient inspired him with an affection, which was met by the gratitude and respect of him whose life had been preserved, and they continued friends up to the time of the Doctor's death, in 1817. I well recollect the interest with which, forty years ago, he often spoke of this event; and an aged and accurate lady of Louisville,* has lately confirmed and extended his narrative.† But Dr. Allison

---

*Mrs. Alexander Pope.

†I have given this fact, which properly belongs to Dr. Allison's biography, with the greater detail, because it serves to illustrate the condition of the early immigrants into the West; and having thus spoken of Louisville, I cannot refrain from expressing a regret, that no one has undertaken to give any account of her early physicians; and still more that there is no longer any one of them living, who could do it, either from his own recollections, or those of the old inhabitants, very few of whom now remain. The same remark is applicable to Pittsburg, Lexington, and St. Louis. In Nashville, it might be done by Dr. Felix Robertson, the first-born of that city, who was a writer for Barton's Journal, in 1804 or '5; and it may be hoped that he will undertake it.

The history of a nation is not to be read in the lives of its generals had a taste for agriculture, as well as surgery, and in 1799, as I have said, he removed to a new farm on the east fork of the Little Miami. In 1805, he returned to the city, where he resumed his practice, and continued it, residing on the south-east corner of Fourth and Sycamore, till his death, on the 22d of March, 1816, at the age of fifty-nine. During his second residence, I lived his neighbor, and saw much of him. Though not profound in science, he was sagacious, unassuming, amiable and kind. As he was the first physician of Cincinnati; resided in or near it 27 years, and was the first who died within its limits, he may be called the *Father of our Profession;* and, as such, I regret that I have not the means of a fuller illustration of his character. When a young physician, I occasionally extracted from him a fact or observation of an important kind, and might have collected many more, if I had then foreseen the interest with which this occasion would invest them. Perhaps what I have said may bring to light some additional information.

The notice of Dr. Sellman is less complete.

Dr. John Sellman was born in the city of Annapolis, Maryland, A. D., 1764. Belonging to one of the ancient and respectable families of that State, he had received a good preparatory education. He entered the army as a surgeon's mate, and reached Cincinnati with Gen. Wayne in the spring of 1793. Where he had practiced his profession previously to that date, I have not been able to learn. The Indian war being over, he resigned in 1794, and took up his residence on Front street, between Sycamore and Broadway; where he continued to reside and practice his profession, till he died in the autumn of 1827,

---

and politicians merely, but comprehends, as its necessary elements, the history of all *classes* of the people, and all *branches* of intellectual, moral, religious, and physical industry. It is high time that our own profession began to look to its rights and duties in this matter; genius, learning, and active beneficence should not be overlooked because they were directed to the preservation, instead of the destruction, of human life.

at the age of sixty-three. Early in the present century the government established the arsenal and barracks in Newport, and Dr. Sellman was employed for many years, as its citizen surgeon. Such a recall, shows that while in the service, he might have discharged his duty faithfully. He was not a graduate ; and without attainments in medicine or the associate sciences above the average of the time at which he was educated, his native good sense and high, gentlemanly bearing, secured to him a large proportion of the best practice of the town; bnt like his contemporary Dr. Allison he did not leave behind him any record of his experience.

Before proceeding to extract his account of Dr. Goforth, his preceptor, we give his sketch of the third physician engaged in practice in Cincinnati at the beginning of the century.

Dr. John Cranmer was a native of Pittsburg. Employed about the office of Dr. Bedford, a distinguished physician of that borough, as it then was, he acquired some knowledge of the symptoms of disease and the properties and doses of medicines; the latter of which he kept in a table drawer, at his residence between Main and Walnut streets, on the north side of Second, for some time after his emigration in 1798. It is worthy of remark, that from this humble beginning, and without original education, or the study of medical books, subsequently he attained to a position of considerable personal and some professional respectability; supporting his family by his practice and continuing to advance in reputation up to the time of his death, which occurred from cholera in 1832. His age I do not know, but as he came here a man, he could not have been less than fifty-five or 'six. He died the latest of all the physicians not of the army, who practiced here in the last century and consequently the whole have been dead about twenty years.

Dr. Drake entered the office of Dr. Goforth in 1800. He thus describes his preceptor:

Dr. William Goforth, of whom I know more than of all who have been mentioned, was born in the city or town of New York, A. D., 1766. His preparatory education was what may be called tolerably good. His private preceptor was Dr. Joseph Young, of that city, a physician of some eminence, who, in the year 1800, published a small volume on the universal diffusion of electricity, and its agency in astronomy, physiology and therapeutics, speculations which his pupil cherished throughout life. But young Goforth, also, enjoyed the more substantial teachings of that distinguished anatomist and surgeon, Dr. Charles McKnight, then a public lecturer in New York. In their midst, however—A. D., 1787–8—he and the other students of the forming school of that city, were dispersed by a mob, raised against the cultivators of anatomy. He at once resolved to accompany his brother-in-law, the late Gen. John S. Gano, into the West; and on the 10th of June, 1788 landed at Maysville, Kentucky, then called Limestone. Settling in Washington, four miles from the river, then in population the second town of Kentucky, he soon acquired great popularity, and had the chief business of the county for eleven years. Fond of change, he determined then to leave it; and in 1799 reached Columbia, where his father, Judge Goforth, one of the earliest and most distinguished pioneers of Ohio, resided. In the spring of the next year, 1800, he removed to Cincinnati, and occupied the Peach Grove House, vacated by Dr. Allison's removal to the country. Bringing with him a high reputation, having an influential family connection, and being the successor of Dr. Allison, he immediately acquired an extensive practice. But without these advantages he would have gotten business, for, on the whole, he had the most winning manners of any physician I ever knew, and the most of them. Yet they were all his own, for in deportment he was quite an original. The painstaking and respectful courtesy with which he treated the poorest and humblest people of the village, seemed to secure their gratitude; and the more especially as he dressed with precision, and never left his house in the morning till his hair was powdered by our itinerant barber, John Arthurs, and his gold headed cane was grasped by his gloved hand. His kindness of heart was as much a part of his nature, as hair powder was of his costume; and what might not be given through benevolence, could always be extracted by flattery, coupled with professions of friendship, the sincerity of which he never questioned. In conversation he was precise yet fluent, and abounded in anecdotes, which he told in a way that others could not imitate. He took a warm interest in the politics of what was then the North Western Territory, being at all times the earnest advocate of popular rights. His devotion to Masonry, then a cherished institution of the village, was such that he always embellished his signature with some of its symbols. His hand writing was peculiar, but so remarkably plain, that his poor patients could read it without difficulty, and felt flattered to think he should have taken so much pains in writing for *them.* In this part of his character, many of us might find a useful example.

To Dr. Goforth the people were indebted, for the introduction of the Cow-pock, at an earlier time, I believe, than it was elsewhere naturalized in the West. Dr. Benjamin Waterhouse, of Boston, had received infection from England, in the year 1800, and early in 1801, Dr. Goforth received it, and commenced vaccination in this place. I was myself one of his first patients, and seeing that it has extended its protecting influence through fifty years, I am often surprised to find medical gentlemen shying off from a case of small-pox.

At the time Dr. Goforth was educated in New York, the writings of Dr. Cullen had not superseded those of Boerhaave, into whose system he had been inducted. Yet the captivating volume of Brown had fallen into his hands, and he was so far a Brunonian, as to cherish an exceeding hostility to the copious depleting practice of Dr. Rush, which came in vogue the beginning of this century. In fact he would neither buy nor read the writings of that eminent man. Yet his practice was *not* that of Brown; though it included stimulants and excluded evacuants, in many cases, in which others might have reversed those terms. In looking back to its results, I may say, that in all, except the most acute forms of disease, his success was creditable to his sagacity and tact.

Fond of schemes and novelties, in the spring of the year 1803, at a great expense, he dug up, at Bigbone lick, in Kentucky, and brought away the largest, most diversified, and remarkable mass of huge fossil bones, that were ever disinterred at one time or place in the United States; the whole of which he put into the possession of that swindling Englishman, Thomas Ashe, alias, Arville, who sold them in Europe and embezzeled the proceeds.

Dr. Goforth was the special patron of all who, in our olden time, were engaged in searching for the precious metals in the surrounding wilderness. They brought their specimens of pyrites and blende to him, and generally contrived to quarter themselves on his family, while he got the requisite analyses made by some black or silver smith. In these researches, Blennerism or the turning of the forked stick, held by its prongs, was regarded as a reliable means of discovering the precious metals not less than water. There was, also, in the village, a man by the name of Hall, who possessed a glass through which he could see many thousand feet into the earth; a feat which I think has not been surpassed, by any of those whom our modern Cincinnati has *feted* for their clairvoyance.

The clarification of ginseng and its shipment to China was, at the beginning of this century, a popular scheme, in which the Doctor eagerly participated; but realized by it much less than those who have since extracted from that root an infallible cure for tubercular consumption.

This failure however did not cast him down; for about the time it occurred, the genuine East India Columbo root was supposed to be discovered in our surrounding woods; and he immediately lent a hand to the preparation of that article for market. It turned out, however, to be the *Frasera verticillata,* long known to the botanist, and essentially distinct from the oriental bitter.

While these various projects were keeping the Doctor's imagination in a state of high and pleasurable excitement, he became enamored with the Mad River country; to which in the very infancy of its settlement he made a winter visit. Beyond where Urbana has been since built, was the Indian village of Mechacheck, at which he arrived at night, expecting to find inhabitants, but found none; being without the means of kindling a fire and unable to travel back in the dark he came nigh perishing from the cold. Subsequently he made another visit in the month of June, and took me with him. It required five days to reach King's Creek, a few miles beyond the present Urbana, which then had one house and Springfield another. The natural scenery after passing the village of Dayton, was of such exquisite beauty that I was not surprised at the Doctor's fascination; but a residence there was not in store for him—he had a different destiny.

The time at length arrived when young Cincinnati was to lose the most popular and peculiar physician which had appeared in the ranks of her infant profession, or indeed has *ever* belonged to it, and the motives and manner of the separation were in keeping with his general character. The French Revolution of 1789, had exiled many educated and accomplished men and women, several of whom found their way into the new settlements of the West. The Doctor's political sympathies were with the Revolutionists, but some of the exiles reached the town of Washington where he resided, and their manners and sufferings triumphed over his repugnance to aristocracy; till pictures of the beauty and elegance of French society began to fill his imagination. Thus impressed he came to Cincinnati, where Masonry soon made him acquainted with an exiled lawyer of Paris; who resided on the corner of Main and Third street, where the banking edifice of the Trust Company now stands. This gentleman, M. Mennesier, planted the vineyard of which I have spoken, and carried on a bakery in the lower story of his house, while the upper was the lodge of Nova Cesarea Harmony No. 2. The Doctor's association with this member of the *beau monde* of course raised his admiration for Gallic politeness still higher; and just at the time when he began, in feeling, to prefer French to Anglo-American society, President Jefferson purchased Louisiana from Buonaparte, first consul of the *republique francaise.* The enchanting prairies of Mad River were now forgotten, and he began to prepare for a southern migration. Early in the spring of 1807, he departed in a flat boat for the coasts and bayous of the lower Mississippi; where he was soon appointed a parish judge, and subsequently elected by the Creoles of Attacapas to represent them in forming the first Constitution of the State of Louisiana; soon after which he removed to New Orleans. During the invasion of that city by the British, he acted as surgeon to one of the regiments of Louisiana volunteers. By this time his taste for French manners had been satisfied, and he determined to return to the city which he had left in opposition to the wishes of all his friends and patients. On the first of May, 1816, he left New Orleans, with his family, on a keel boat; and on the 28th of the next December, after a voyage of eight months, he reached our landing. He immediately reacquired business; but in the following spring he sunk under hepatitis, contracted by his summer sojourn on the river.

In reference to the mortality of the medical men of Cincinnati, Dr. Drake remarks, that twenty-five times as many died in the second as during the first half of its history. This, he thinks, indicates not only a great increase in the medical population, but also an increased ratio of mortality. Most of the early physicians attained to an age somewhat advanced, the average of the six who practiced there the longest having been fifty-six years. But of those who died in the second half very few were far advanced, and many were young. He adds, that "it is truly a sad thing, that in the United States the progress of civilization and science should so violate the laws of health, as to shorten the lives of those who are laboring to promote it."

Dr. Drake further remarks concerning the pioneers of the profession of Cincinnati, that "none of them had graduated, and the majority had, probably, not even attended a single course of the imperfect lectures which were delivered in the half-organized schools in the last century."

The following is his graphic and amusing sketch of medical practice and medical study in the Queen city half a century ago.

Every physician was then a country practitioner, and often rode twelve or fifteen miles on bridle paths to some isolated cabin. Occasional rides of twenty and even thirty miles were performed on horse back, on roads which no kind of carriage could travel over. I recollect that my preceptor started early, in a freezing night, to visit a patient eleven miles in the country. The road was rough, the night dark, and the horse brought for him not [as he thought] gentle; whereupon he dismounted after he got out of the village, and, putting the bridle into the hands of the messenger, reached his patient before day on foot. The ordinary charge was twenty-five cents a mile, one-half being deducted, and the other paid in provender for the horse, or produce for the family. These pioneers, moreover, were their own bleeders and cuppers, and practiced dentistry, not less, certainly, than physic—charging a quarter of a dollar for extracting a single tooth, with an understood deduction if two or more were drawn at the same time. In plugging teeth, tin-foil was used instead of gold-leaf, and had the advantage of not showing so conspicuously. Still, further, for the first twelve or fifteen years, every physician was his own apothecary, and ordered little importations of cheap and inferior medicines by the dry goods merchants once a year, taking care to order them long before they were needed. Mr. James Ferguson, a volunteer in Harmer's campaign, began mercantile business near the corner of Third and Sycamore streets, in 1792, about sixty years ago. The only road to Philadelphia was then through Lexington, Danville and Crab Orchard to Cumberland gap, nearly south, across the broadest part of Kentucky; then north-east, through Abingdon, Staunton, and Winchester, Virginia, by Baltimore, to the city which supplied us with medicines, not less than every other article of merchandize. As he lately informed me, from twenty-five to thirty days were the required time to transfer them from Philadelphia to Brownsville, and as much more by the river to Cincinnati. Thus from four to five months were required for the importation of medicine, which, at this time, being ordered by telegraph and sent by express, may be received in two days, or a sixtieth part of the time. Thus science has condensed minutes into seconds. The prices at which these medicines were sold, differed widely from those of the present day. Thus an emetic, a Dover's powder, a dose of Glauber's salt, or a night draught of paregoric and antimonial wine, *haustus anodynus* as it was learnedly called, was put at twenty-five cents, a vermifuge or blister at fifty, and an ounce of Peruvian bark at seventy-five for pale, and a dollar for best red or yellow. On the other hand personal services were valued very low. For bleeding twenty-five—for sitting up all night a dollar, and for a visit from twenty-five to fifty cents, according to the circumstances or character of the patient.

The physician often carried medicines in his pocket, and dealt them out in the sick room; but the common practice was to return home, compound and send them out. It was my function during the first three years of my pupilage thus to put up and distribnte medicines over the village; in doing which I was occasionally brought as far, or even further, west than the spot on which we are now assembled. In this distribution when my preceptor was, I may say, principal physician, fleetness was often necessary to the safety of patients; and as there were no pavements, the shortest way through a mud-hole, seemed to boyish calculation the best, and although half a century has rolled away, such is the influence of early impressions, that I have not yet gotten rid of the conviction; and having thus distinctly obtruded myself on your notice, I must continue in it to the end, for the purpose of saying a word on medical education in those early times.

Beginning on the 20th of December, 1800, at Peach Grove where the Lytle house now stands, my first assigned duties were to read Quincy's Dispensatory and grind quicksilver into *unguentum mercuriale;* the latter of which, from previous practice on a Kentucky hand mill, I found much the easier of the two. But few of you have seen the genuine, old Doctor's shop of the last century; or regaled your olfactory nerves in the mingled odors which, like incense to the God of physic, rose from brown paper bundles, bottles stopped with worm-eaten corks, and open jars of ointment, not a whit behind those of the apothecary, in the days of Solomon; yet such a place is very well for a student. However idle, he will be always absorbing a little medicine; especially if he sleeps beneath the greasy counter. But I must leave off philosophising and return to my narrative. New studies and a new *studio* awaited me; and through the ensuing spring and summer the adjoining meadow with its forest shade trees, and the deep and dark wood of the near banks and valley of Deer Creek, acted in the manner of the wilderness on the young Indian, caught and incarcerated in one of the school houses of civilization. Underneath those shade trees, the roots of which still send up an occasional scion, or among the wild flowers of the wood, which exhaled incense to Flora instead of Æsculapius, it was my allotted task to commit to memory Cheselden on the bones, and Innes on the muscles, without specimens of the former or plates of the latter; and afterwards to meander the currents of humoral pathology, in Boerhaave and Vanswieten; without having studied the chemistry of Chaptal, the physiology of Haller, or the Materia Medica of Cullen.

Such was the beginning of medical education in Cincinnati. I say *beginning*, for I was its *first* pupil. I have already mentioned the aversion of my preceptor to the system of Dr. Rush; which went so far, that he forbade my reading the few writings of that gentleman which he happened to have. The new publications, brought out by Dr. Stites, were, however, a temptation to disobedience not to be resisted; and before my master was aware of the fact, I commanded his respect, by knowing things of which his prejudices had kept him ignorant. You will smile when I tell you that a consequence of this was, that he who never saw defects in one he loved, soon after this—in 1803—began to ask my opinion on cases which he took me with him to see. In the spring of the following year, he made me his partner; and in the summer of the next, when I was preparing to visit the University of Pennsylvania, he favored me with an autograph diploma, setting forth my ample attainments, in all the branches of the profession; and subscribing himself, as he really was, Surgeon General of the First Division of Ohio Militia. This was undoubtedly the first medical diploma ever granted in the interior valley of North America. I cherish it as a memorial of olden time, and still more as the tribute of a heart so generous, as to set aside all the dictates of judgment, on the qualifications of the stripling to whom it was spontaneously given. By *its* authority I practiced medicine for the next eleven years; when it was corroborated by another from the University—the first ever conferred, by that or any other school, on a Cincinnati student.

And now, having successively presented myself, as the first pupil and the first graduate of the town, I may add, as completing the medical history of its twenty-one years, that I was also its first medical author; having in 1809, written for distribution, a pamphlet on its etiology and diseases, under the title of "Notices Concerning Cincinnati." It was printed in the office of the Rev. John W. Browne, and set up by a worthy man, at this moment, perhaps, among you, Mr. Sacket Reynolds, then an apprentice in the office. I cannot say of it as Professor Woodhouse said of his introductory lecture after delivering it the twelfth time, that I find new beauties in it every year; yet we must not entirely everlook the "day of small things." For this is the *only* specimen of our medical literature, for the first half of our city and professional existence.

I have thus, gentlemen, given you some account of our past—of the first third part of the life-time of our city profession. It was then in its infancy, and the footprints of childhood soon grew dim. Yet, if possible, they should not be allowed to fade away. Under this sentiment, I have used a little, the chisel of Old Mortality; and feel that I have but discharged a duty which at all times, the present owes to the past; and the members of a generous profession to each other. Having been *pars minima* of what I have described, the verity of history reqnired that I should not exclude myself.

We have room at present, for but a single other extract, which, like the last, is illustrative of the early times in the West, when our author was a student of medicine.

Between Second and Third Streets, near where we now have the eastern end of the market-house, there was a single frame tenement, in which I lived with my preceptor in 1805. In a pond, directly in front, the frogs gave us regular serenades. Much of the square to which this house belonged was fenced in, and served as a pasture ground for a pony which I kept for country practice; and of which, as illustrative of the West at that time, I must say a word. He was a Chickasaw, that is, an Indian horse, from what is now the north-eastern corner of the State of Mississippi. The boatmen of that day had to return from New Orleans by land. They generally began the journey on foot, but purchased horses of the Indians when they got tired of walking. Thus Chickasaw and Choctaw ponies were not uncommon in this region at that time. It was a pecularity of mine, (perhaps of all the Indian horses, as it would be a protection from thieves,) that he could not be caught after night-fall; and this reminds me of a professional case, still further illustrative of our olden time. Witches were not then extinct, and some of them were actually known. One of the most mischievous lived a few miles back in the country, and bewitched a woman on the river bank. Her husband came at dusk for assistance, and went into the lot to assist in catching my horse, which, of course, we failed to do, and he ascribed the failure to the witch having entered the animal. It only remained to give him a paper of medicine, which he afterwards assured me was the best he had ever tried, for, as he entered the door of his cabin, the witch passed out through the small back window, and escaped up the steep hill into the woods. He carefully preserved the medicine as a charm, and found it more efficacious than a horse-shoe nailed over the door, which, before the united skill of Dr. Goforth and myself had been brought to bear on this matter, was the most reliable counter-charm.

These Discourses were written in the midst of pressing professional duties and in the intervals of other literary labors, and the reader will not fail to discover in them the marks of hasty composition; but this will be overlooked and forgotten in the spirit and interest of the narrative, the excellence of the matter, and the elevation and liberality of sentiment pervading them. We hope they are the beginning of a series of such biographical memoirs, and that we shall have many opportunities of enlivening our pages with sketches of the pioneer physicians of the West. Lexington, St. Louis, Nashville, New Orleans, and Louisville, to say nothing of our smaller towns and villages, have their medical history, which ought to be written. True, not many of them can point among their surviving practitioners to one whose memory, as in this instance, runs back to the period of their foundation; but this constitutes no valid reason why all that can now be gathered up of their early annals should not be recorded and preserved. Dr. Drake, we believe, has but one analogue in all our western cities. There is but one physician now living in all the region which fifty years ago formed the backwoods of America, who has seen the city of his birth pass through the changes of more than half a century. Dr. Felix Robertson, of Nashville, already referred to in an extract from these discourses, was born in that city, and, we believe, was the first male child born in the settlement. He was reared and educated there, and there in the laborious practice of physic, he has passed a long and singularly amiable and blameless life. His recollections must embrace the earliest physicians of Nashville, and his strong sense of justice, and great amiability of temper would enable him to estimate impartially the merits of the many professional competitors whom he has seen, one after another, called away from their labors. It is to be hoped that this venerable and revered gentleman may be induced to set at once about writing the medical history of his native city; and others, we trust, will be stimulated by the example of Dr. Drake to contribute to the stock of our biographical literature.

Our limits are exhausted and we must reserve our comments upon the second discourse for a future number. Its topics are weighty, and their discussion is not to be entered upon without care and reflection. "The origin and influence of medical periodical literature, and the benefits of public medical libraries," are the subjects of this discourse.

